# Preprocedural cardiac computed tomography versus transesophageal echocardiography for planning left atrial appendage occlusion procedures

**DOI:** 10.1186/s44348-024-00029-y

**Published:** 2024-09-04

**Authors:** Bing Wei Thaddeus Soh, Carlos Sebastian Gracias, Wee Han Sim, Michael Killip, Max Waters, Kevin P. Millar, Julie M. O’Brien, Thomas J. Kiernan, Samer Arnous

**Affiliations:** 1 Department of Cardiology, University Hospital Limerick, Limerick, Ireland; 2 Department of Radiology, University Hospital Limerick, Limerick, Ireland; 3 School of Medicine, University of Limerick, Limerick, Ireland

**Keywords:** Atrial fibrillation, Left atrial appendage closure, Multidetector computed tomography, Transoesophageal echocardiography

## Abstract

The heterogeneous anatomy of the left atrial appendage (LAA) necessitates preprocedural imaging essential for planning of percutaneous LAA occlusion (LAAO) procedures. While transoesophageal echocardiography (TOE) remains the gold standard, cardiac computed tomography (CT) is becoming increasingly popular. To address the lack of consensus on the optimal imaging modality, we compared the outcomes of preprocedural TOE versus CT for LAAO procedure planning. A retrospective single-center cohort study of all LAAO procedures was performed to compare the outcomes of patients receiving preprocedural TOE versus those receiving CT. The primary outcome was procedural success and rate of major adverse events. The secondary outcomes were total procedure time, rate of device size change, and maximum landing zone diameter. A total of 64 patients was included. Of these, 25 (39.1%) underwent TOE and 39 (60.9%) underwent CT. There was no significant difference in the procedural success rate (96.0% vs. 100%, *P* = 0.39) or major adverse event rate (4.0% vs. 5.1%, *P* > 0.99) between TOE and CT patients. Compared with TOE, CT was associated with significantly shorter median procedure time (103 min vs. 124 min, *P* = 0.02) and a lower rate of device size change (7.7% vs. 28.0%, *P* = 0.04). Compared to CT, TOE was associated with a significantly smaller mean maximum landing zone diameter (20.8 mm vs. 25.8 mm, *P* < 0.01) and a higher rate of device upsizing (24.0% vs. 2.6%, *P* = 0.01). No significant difference in detected residual leak rates was found between TOE and CT (50.0% vs. 52.2%, *P* > 0.99). Planning of LAAO procedures with CT is associated with a shorter total procedure time and a lower rate of device size change and is less likely to underestimate the maximum landing zone diameter.

## Background

The left atrial appendage (LAA) is a highly complex and heterogeneous structure implicated in the pathogenesis of nonvalvular atrial fibrillation (AF)-related strokes [1]. Combined with a multilobed and irregular anatomy, the LAA experiences slow flow due to the absence of atrial systole during AF and predisposes patients to thrombus formation, increasing the risk of ischemic stroke by a factor of five [2]. Although long-term oral anticoagulation (OAC) remains the standard of care for stroke prevention in AF patients, percutaneous LAA occlusion (LAAO) may be considered in select AF patients who have an absolute or relative contraindication to long-term OAC [2, 3].

While the real-world efficacy and periprocedural safety of percutaneous LAAO have been well-documented in the form of long-term outcomes of randomized controlled trials and LAAO registries, the evidence and consensus on the optimal preprocedural imaging modality for LAAO procedure planning are weaker [4–6]. From its origins as the sole imaging modality in early pivotal trials, two-dimensional (2D) transoesophageal echocardiography (TOE) is widely regarded as the gold standard for LAAO procedures [7–9]. However, cardiac computed tomography (CT) is becoming increasingly popular due to its ability to capture high-resolution, 3D images noninvasively. CT provides consistently accurate LAA measurements regardless of elliptical ostial morphology or left atrial load [9, 10].

Accurate preprocedural measurement of the dimensions of the LAA is important for accurate device sizing, procedural efficiency, and preventing complications from peridevice leakage or device embolization. Observational studies have found that CT is superior to TOE for planning LAAO procedures, resulting in less frequent device undersizing, more accurate LAA measurements, and fewer residual leaks [11–14]. However, the real-world impacts of using CT for more accurate LAA assessments compared with TOE are less well understood in everyday clinical settings.

Given the lack of an established consensus on the optimal imaging modality for the planning of LAAO procedures, we compared our clinical experiences with preprocedural TOE and CT for percutaneous LAAO.

## Methods

### Study design and population

We undertook a retrospective single-center cohort study of patients who underwent percutaneous LAAO using TOE or CT for preprocedural planning. All patients who underwent preprocedural imaging prior to percutaneous LAAO between January 2018 and December 2022 were included in the study. Included patients with a clinical indication of percutaneous LAAO were fully informed about the procedure and provided informed written consent for the procedure. Preprocedural TOE or CT is routinely performed in the outpatient setting. TOE served as the default modality until January 2022, when CT became the default choice. Clinical considerations also played a minor role in assigning patients to both TOE (severe renal impairment and/or inability to tolerate contrast administration) and CT (known esophageal stricture and/or inability to tolerate TOE). On the day of the procedure, TOE was performed to rule out LAA thrombus, a contraindication for the procedure. While LAA measurements were performed on the same day as TOE, they were used for correlation purposes with CT but not for guiding the clinical selection of LAAO device size.

Patients were assigned to the TOE group if they underwent preprocedural TOE only and to the CT group if they underwent preprocedural CT. The impact of preprocedural imaging modality on a percutaneous LAAO procedure was assessed by collecting and analyzing key procedural characteristics and outcomes. The primary outcomes were procedural success and rate of major adverse events, which were defined as a composite of periprocedural stroke, death, device embolism, pericardial effusion, myocardial infarction, and surgical conversion. The secondary outcomes consisted of variables of procedure characteristics: total procedure/fluoroscopy time, rate of change of device size, and maximum landing zone diameter. A post hoc assessment was performed using postprocedural CT to evaluate residual leaks after LAAO in both groups.

### Transesophageal echocardiography

**Fig. 1:**
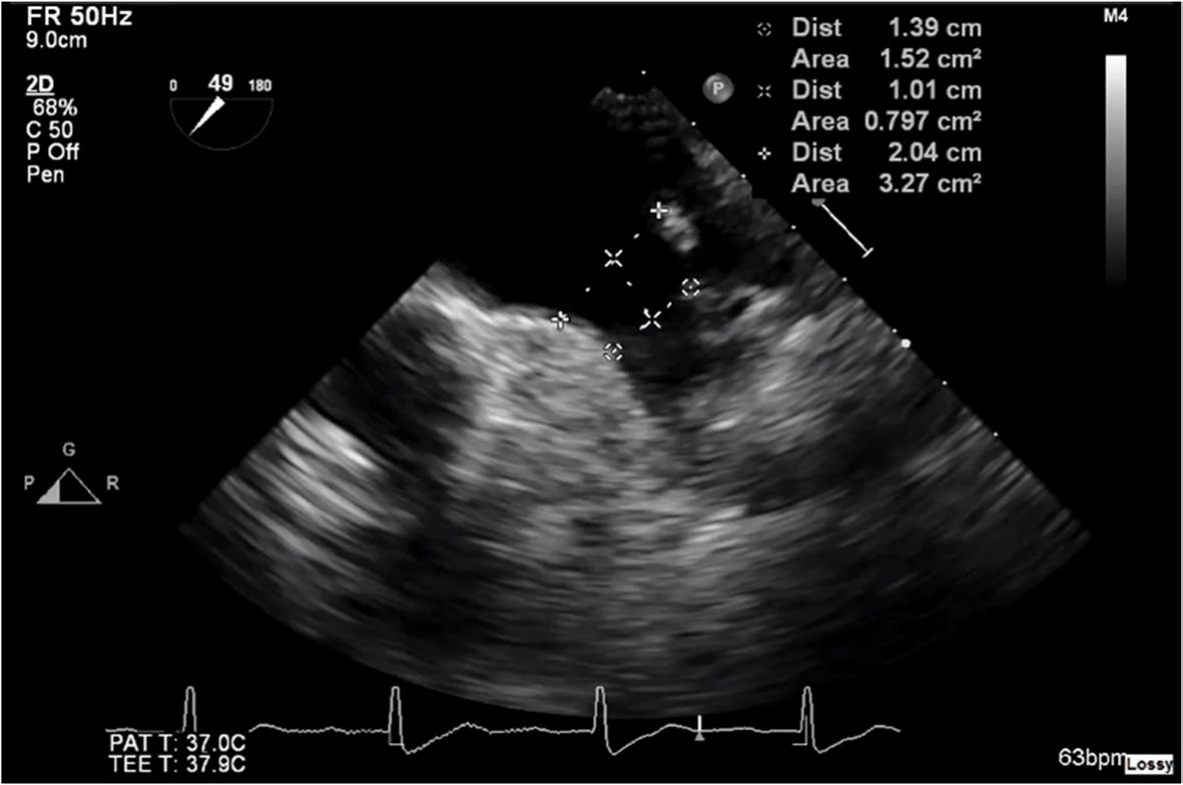
Transesophageal echocardiography measurement of left atrial appendage dimensions at a 49° angle at end-systole and in alignment with the level of the coumadin ridge

Preprocedural TOE was performed in 2D mode using a Phillips EPIQ CVx cardiovascular ultrasound apparatus with an X7-2 T ultrasound transducer probe (Philips). LAA size, morphology, and presence of thrombus were systematically evaluated along with interatrial septum characteristics to guide transseptal puncture. Following stepwise obtainment of informed consent, a topical 10% lidocaine spray was administered to the posterior oropharynx, preoxygenation, and intravenous midazolam or diazepam was administered incrementally to sedate the patient. The transducer probe was advanced to the midesophageal level to visualize the LAA in a two-chamber view. The LAA was then thoroughly assessed at angles of 0°, 45°, 90°, and 135° to evaluate ostial and landing zone widths in accordance with manufacturer recommendations for an Amplatzer Amulet LAAO device (Abbott). The maximal landing zone dimension was subsequently measured 10 to 12 mm from the LAA ostium at end-systole, in anatomical alignment with the origin of the left circumflex artery and the left superior pulmonary vein ridge (Fig. 1). Device selection was then performed by aligning the obtained measurements with the manufacturer’s sizing chart.

### Cardiac computed tomography

**Fig. 2:**
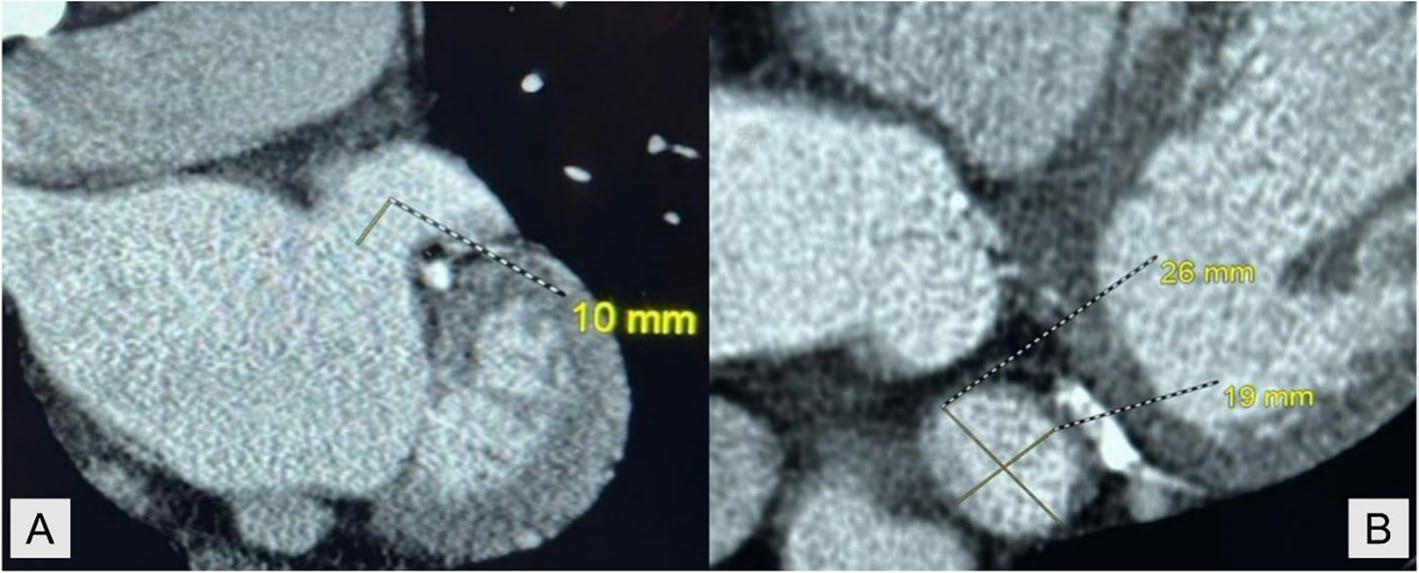
Computed tomography measurement of left atrial appendage (LAA) dimensions. **A** Coronal projection of the LAA with maximal craniocaudal depth from the LAA ostium. **B** Axial projection of the LAA with maximal landing zone diameter

Preprocedural CT imaging was performed with prospective electrocardiogram gating using a SOMATOM Definition AS 128-slice CT scanner (Siemens). A target heart rate of < 65 beats/min was achieved by incremental dosing of oral and/or intravenous metoprolol. LAA dimensions were measured at end-diastole to maximize LAA volumes. Images were acquired craniocaudally with a gantry rotation time of 330 ms, a tube potential of 100 kV, a tube current of 300 to 500 mA, and detector collimation of 128 × 0.6 mm. Arrhythmia detection software was used to minimize the need for scanning during atrial and ventricular ectopy. Following a chest topogram, an ulrichINJECT syringeless CT motion system (GE Healthcare) was used to administer Omnipaque 350 contrast (GE Healthcare) through an 18-gauge high-flow intravenous cannula. After a 20 mL test bolus and 13-s delay, contrast-enhanced scanning was initiated with a trigger threshold of 150 Hounsfield units (HU) and the ascending aortic root was chosen as the region of interest. This was followed by injection of 70 mL of contrast, followed by a 40 mL 1:1 mixed contrast-saline chaser. Contrast-phase images were reconstructed using slices 0.5 mm thick at increments of 0.25 mm, while noncontrast phase images were reconstructed using slices 3 mm slice thick at increments of 3 mm. All images were analyzed using the commercially available OsiriX DICOM Viewer software package (Pixmeo SARL). The 3D multiplanar reconstruction was achieved with 90° locking-in of the sagittal, coronal, and axial planes (Fig. 2). LAA size and morphology and presence of thrombus were assessed.

The LAA ostium was identified using the left circumflex artery and the coumadin ridge as landmarks. Once the angle of the plane was set, the landing zone was located 10 mm from the LAA ostium. At this point, both the maximal and minimal landing zone dimensions were recorded. While recent studies have shown that a perimeter-derived diameter of the landing zone on CT imaging provides a more accurate estimate for LAAO device sizing [10], our center used the established parameter of maximal dimension to size the LAAO device, following recommendations. This approach remains the official standard of practice as determined by expert consensus [15].

### Percutaneous LAAO procedure

All percutaneous LAAO was performed under conscious sedation, using fluoroscopy and intracardiac echocardiography (ICE) for intraprocedural guidance. In patients on OAC, anticoagulation was discontinued 48 h prior to the procedure. Access was obtained through the right femoral vein using two sheaths to accommodate the ICE catheter and the LAAO device delivery system. Following transseptal puncture, a stiff guidewire was advanced into the left atrium (LA) and the delivery sheath was advanced over the stiff guidewire to dilate the interatrial septum, allowing passage of the ICE catheter into the LA. Within the LA, the ICE catheter was positioned either in the left superior pulmonary vein or the mid LA to provide guidance for positioning of the LAAO device in the designated landing zone. The Amplatzer Amulet LAAO device (Abbott) was advanced out of the delivery sheath into the lobe landing zone position. The disc was deployed while the ICE catheter verified orientation and stability. Subsequently, an LAA angiogram was conducted to ensure proper alignment of the device with the LAA neck. Once the operator had assessed the device for leaks, performed a tug test, and confirmed the final position, the delivery system was uncoupled from the LAAO device and retracted.

### Residual leak assessment using postprocedural CT

All patients included in the study were evaluated retrospectively for suitability for postprocedural cardiac CT to assess residual leaks after LAAO. Residual leak (patency) within the LAA was defined as persistent contrast opacification of the LAA from the left atrium. The presence of contrast was assessed quantitatively by measuring the average linear attenuation coefficient (in HU) in the LAA distal to the implanted LAAO device. Using a circle 2 mm in diameter as the region of interest, the LAA was classified as patent if the cutoff of 100 HU was exceeded (sealed if ≤ 100 HU), as previously described [16].

### Statistical analysis

All calculations and analyses were performed using IBM SPSS ver. 27 (IBM Corp). Continuous data with normal distributions were reported as mean ± standard deviation, while data with non-normal distributions were reported as median (interquartile range, IQR). Categorical data were reported as number of patients and percentages. Statistical comparisons of continuous and categorical variables were performed using Student t-test, a median test, and Fisher exact test. A P-value of < 0.05 was considered statistically significant. In patients within the CT group who also underwent preprocedural TOE, Pearson correlation analysis was performed, and Bland–Altman analysis was used to assess bias and limits of agreement (LOAs) between the two imaging modalities. As a previous study had identified a discrepancy of two or more device size intervals as indicative of disagreement, a 6-mm difference was considered clinically significant [17].

## Results

### Baseline patient characteristics

A total of 64 patients underwent percutaneous LAAO with preprocedural imaging in our center. Of these, 25 patients (39.1%) underwent preprocedural TOE and 39 (60.9%) underwent CT, and were subdivided into the respective groups. Baseline patient characteristics of the TOE and CT groups are described in Table 1. The mean age of the cohort was 76.9 ± 5.5 years, with patients in the CT group being significantly older than those in the TOE group (74.8 ± 5.8 years vs. 78.2 ± 5.0 years, *P* = 0.02). A total of 50 male patients (78.1%) was included, with a similar distribution between TOE and CT groups (80.0% vs. 76.9%, *P* > 0.99). Patients in the groups had similar comorbidities, with hypertension and history of ischemic heart disease the two most commonly reported conditions. Patients in the two groups had similar indications for LAAO, with gastrointestinal bleeding (56.3%) being the most common, followed by intracranial hemorrhage (23.4%). The mean CHA_2_DS_2_-VASc scores in the TOE and CT groups were similar (3.6 ± 1.3 vs. 3.9 ± 1.5, *P* = 0.45). The mean HAS-BLED scores in the TOE and CT groups also were similar (3.9 ± 1.0 vs. 4.1 ± 0.9, *P* = 0.31). Mean hemoglobin level was significantly lower in the CT group compared to the TOE group (13.1 ± 1.8 g/dL vs. 12.0 ± 1.7 g/dL, *P* = 0.03).

**Table 1:** Baseline patient characteristics

Characteristic	TOE group (n = 25)	CT group (n = 39)	*P*-value
Patient demographic			
Age (yr)	74.8 ± 5.8	78.2 ± 5.0	0.02
Male sex	20 (80.0)	30 (76.9)	> 0.99
LAAO indication			
Intracranial hemorrhage	6 (24.0)	9 (23.1)	> 0.99
Gastrointestinal bleeding	15 (60.0)	21 (53.8)	0.80
Stroke on anticoagulation	1 (4.0)	3 (7.7)	> 0.99
Epistaxis	1 (4.0)	4 (10.3)	0.64
Hematuria	2 (8.0)	2 (5.1)	0.64
Baseline parameter			
Hemoglobin (g/dL)	13.1 ± 1.8	12.0 ± 1.7	0.03
Creatinine (μmol/L)	85.1 ± 19.5	104.6 ± 68.8	0.12
Left ventricular ejection fraction (%)	53.0 ± 4.8	53.6 ± 2.6	0.57
CHA_2_DS_2_-VASc score	3.6 ± 1.3	3.9 ± 1.5	0.45
HAS-BLED score	3.9 ± 1.0	4.1 ± 0.9	0.31

Values are presented as mean ± standard deviation or number (%)

*TOE* Transesophageal echocardiography, *CT* Computed tomography, *LAAO* Left atrial appendage occlusion

### LAA measurement

**Fig. 3:**
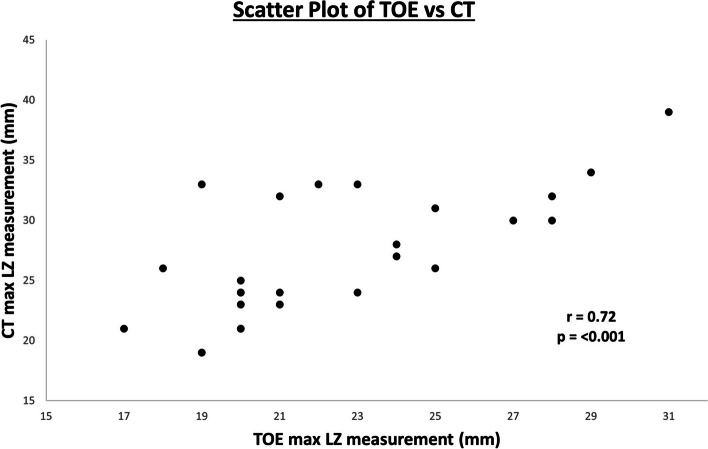
Scatter plot of transesophageal echocardiography (TOE) and computed tomography (CT) measurements of maximum landing zone diameter with Pearson correlation coefficient

**Fig. 4:**
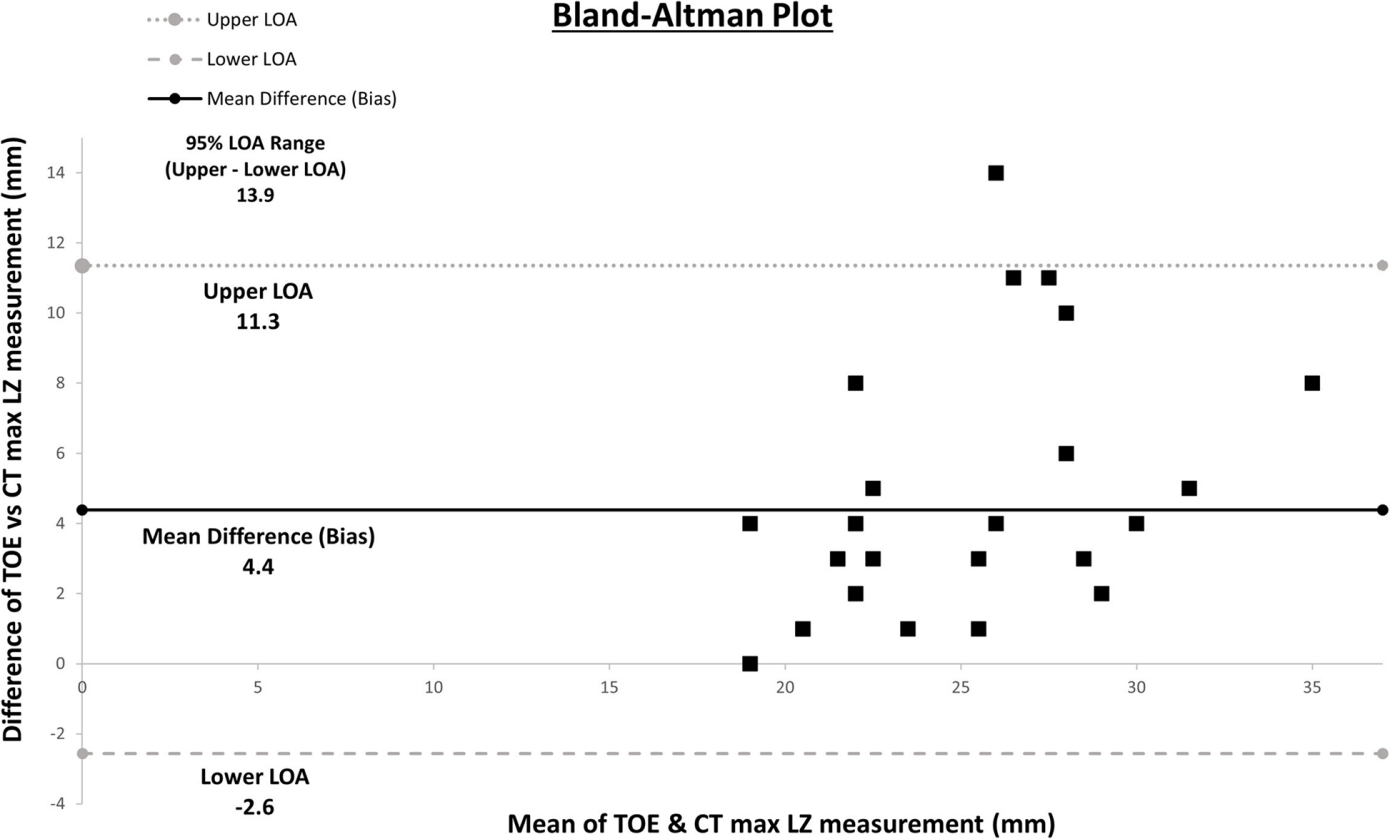
Bland–Altman plot of difference in and mean of transesophageal echocardiography (TOE) and computed tomography (CT) measurements of maximum landing zone diameter. LOA, limit of agreement

The mean maximum landing zone diameter was significantly higher in the CT group compared with the TOE group (20.8 ± 3.0 mm vs. 25.8 ± 5.5 mm, *P* < 0.01). Further correlation and agreement analysis was performed in 28 patients (71.8%) within the CT group who also received preprocedural TOE. As shown in Fig. 3, Pearson correlation identified a strong positive and significant relationship between CT measurements of maximum landing zone diameter and TOE measurements (r = 0.72, *P* ≤ 0.01). Agreement analysis with the Bland–Altman plot revealed a mean difference (bias) of 4.4 mm, 95% LOAs (mean difference ± 1.96 × standard deviation) of 11.3 and –2.6 mm, and a resulting wide 95% LOA range of 13.9 mm (Fig. 4).

### Procedure outcomes and characteristics

Procedure success and major adverse event rates did not differ significantly between the TOE and CT groups (Table 2). Nearly all LAAO procedures resulted in a successfully implanted device in the TOE group (96.0%) and CT group (100%). Of all procedures in the TOE group, only one did not result in successful implantation of an LAAO device. This outcome was attributed to an insufficient maximum landing zone of 14 mm (measured on TOE), which could not accommodate even the smallest Amplatzer Amulet LAAO device. Although the size of the deployed Amplatzer Amulet LAAO devices did not differ significantly between the TOE and CT groups, there was numerically more frequent use of larger devices (31 and 34 mm) in the CT group (23.1% vs. 4.0%, *P* = 0.07). Procedure-related pericardial effusion was the only major adverse event, and it presented with a similar distribution between TOE and CT groups (4.0% vs. 5.1%, *P* > 0.99). While the median fluoroscopy time did not differ significantly between groups (25.5 min [IQR, 19.5–37 min] vs. 27.5 min [IQR, 22–33 min], *P* > 0.99), the median procedure time was significantly shorter in the CT group compared with that of the TOE group (124 min [IQR, 100–175 min] vs. 103 min [IQR, 78–115 min], *P* = 0.02). A change in device size due to poor fit of the initially deployed device was significantly less frequent in the CT group than the TOE group (28.0% vs. 7.7%, *P* = 0.04). Of the device changes, upsizing was significantly more likely to be required in the TOE group (24.0% vs. 2.6%, *P* = 0.01). Device percentage oversizing (percentage difference from measured landing zone size and implanted device size) was significantly higher in the TOE group compared with the CT group (18.6% ± 9.3% vs. 8.7% ± 9.4%, *P* < 0.01).

**Table 2:** Procedure outcomes and characteristics

Characteristic	TOE group (n = 25)	CT group (n = 39)	P-value
Maximum landing zone diameter^a^ (mm)	20.8 ± 3.0	25.8 ± 5.5	< 0.01
Procedure outcome			
Successful device implantation	24 (96.0)	39 (100)	0.39
20 mm	1 (4.0)	1 (2.6)	> 0.99
22 mm	6 (24.0)	12 (30.8)	0.78
25 mm	11 (44.0)	12 (30.8)	0.30
28 mm	5 (20.0)	5 (12.8)	0.49
31 mm	1 (4.0)	5 (12.8)	0.39
34 mm	0 (0)	4 (10.3)	0.15
Major adverse event	1 (4.0)	2 (5.1)	> 0.99
Periprocedural myocardial infarction	0 (0)	0 (0)	-
Periprocedural stroke	0 (0)	0 (0)	-
Device embolism	0 (0)	0 (0)	-
Pericardial effusion	1 (4.0)	2 (5.1)	> 0.99
Surgical conversion	0 (0)	0 (0)	-
Procedure characteristic			
Procedure time (min)	124 (100–175)	103 (78–115)	0.02
Fluoroscopy time (min)	25.5 (19.5–37)	27.5 (22–33)	> 0.99
Device size change	7 (28.0)	3 (7.7)	0.04
Extra device for upsizing	6 (24.0)	1 (2.6)	0.01
Extra device for downsizing	1 (4.0)	2 (5.1)	> 0.99
Device oversizing from measured landing zone (%)	18.6 ± 9.3	8.7 ± 9.4	< 0.01
Postprocedural CT follow-up			
Received post-LAAO CT follow-up	16 (64.0)	23 (59.0)	0.80
Median time to CT follow-up (day)	661.5 (434.5–934.5)	573 (294–896)	0.57
Assessment of LAA patency (residual contrast in the LAA)	8/16 (50.0)	12/23 (52.2)	> 0.99

Values are presented as mean ± standard deviation, number (%), or median (interquartile range). Percentages may not total 100 due to rounding

*TOE* Transesophageal echocardiography, *CT* Computed tomography, *LAAO* Left atrial appendage occlusion, *LAA* Left atrial appendage

^a^LAA sizing characteristic

### Postprocedural residual leak

Postprocedural CT was performed in 16 patients (64.0%) in the TOE group and 23 patients (59.0%) in the CT group (Table 2). The median time to CT follow-up did not differ significantly between the TOE and CT groups (661.5 days [IQR, 434.5–934.5 days] vs. 573 days [IQR, 294–896 days], *P* = 0.57). Within the cohort of patients who underwent postprocedural CT, there was no significant difference in LAA patency (residual contrast leak) between the TOE and CT groups (8 [50.0%] vs. 12 [52.2%], *P* > 0.99).

## Discussion

By comparing our clinical experiences with preprocedural TOE and CT for percutaneous LAAO, our study did not detect any significant differences in procedural outcomes or residual contrast leaks between the two imaging modalities. However, the study did demonstrate that, compared with TOE, preprocedural CT is associated with favorable procedure characteristics, such as shorter procedure time and reduced likelihood of device size change, especially upsizing. While TOE and CT measurements of the LAA landing zone diameter had a strong and significant positive correlation, the wide 95% LOA range of 13.9 mm suggests unacceptable agreement between the two imaging modalities as it translates to a device size discrepancy exceeding two or more intervals (6 mm), a difference that is likely clinically significant. The prevalence of CT-detected residual leaks in our study was consistent with findings reported elsewhere, and while TOE-detected residual leaks are associated with greater thromboembolic risks, the clinical significance of CT-detected peridevice leaks remains unclear [18].

Due to the complexity and heterogeneity of LAA anatomy, LAAO device sizing is challenging for four key reasons:

1. The LAA structure varies significantly between individuals.
2. The shape of the LAA ostium can range from round to elliptical.
3. The LAA ostium is not clearly defined along an anatomical line but an imaginary boundary between the left circumflex artery and the left superior pulmonary vein ridge, leading to significant inter-operator variability in interpretation.
4. The distensible nature of the LAA results in dimensions that are significantly influenced by volume (preload status).

Studies consistently demonstrate that, compared with LAA using 2D-TOE imaging, 3D-CT imaging is associated with larger landing zone measurements [11, 12]. These measurements correlate with more appropriately sized devices, resulting in fewer residual leaks postdeployment [11–14]. The results from our study mirror these findings, with the mean measured maximum landing zone diameter being significantly higher in the CT group compared to the TOE patients. The larger maximum landing zone diameter measurement within the CT group also more closely resembles the true anatomical LAA dimensions, as assessed from the significantly lower likelihood of device size change, and significantly lower percentage device oversizing in the CT group. The tendency for TOE to undersize the LAA is also reflected by the significantly increased likelihood of device upsizing.

**Fig. 5:**
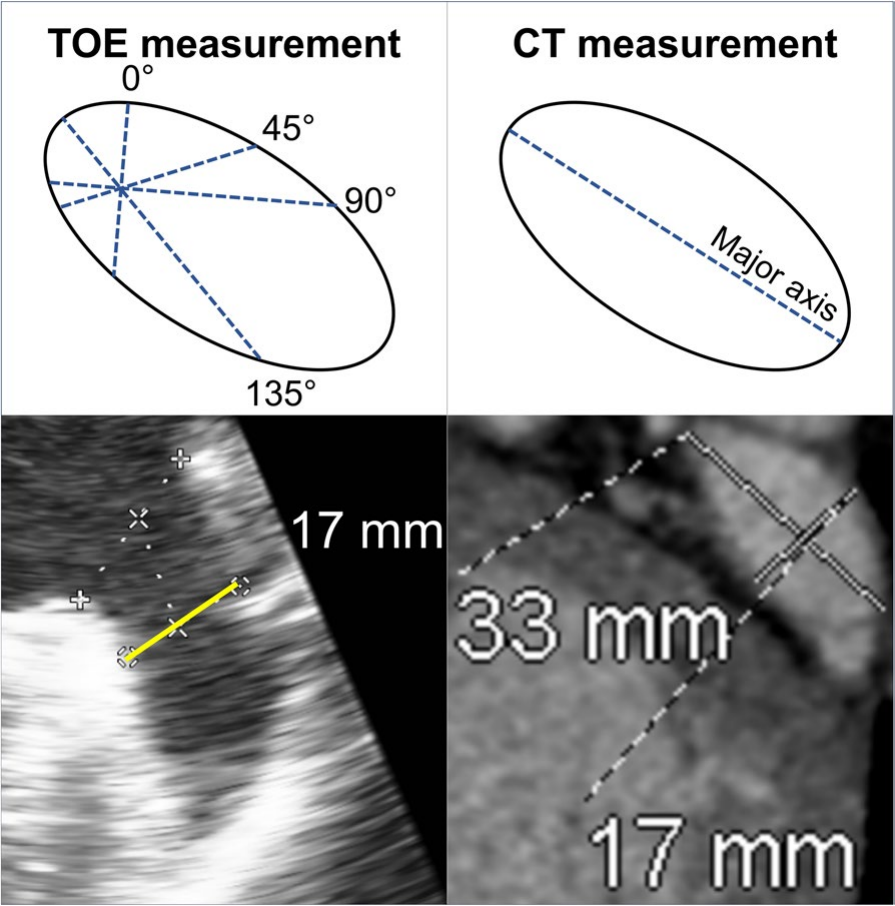
Comparison of transesophageal echocardiography (TOE) and computed tomography (CT) assessments of maximum landing zone diameter in the same patient. Despite measurements from multiple angles, the two-dimensional assessment by TOE may miss the major axis of an eccentric oval-shaped left atrial appendage, resulting in underestimation of the landing zone

The mechanism by which CT more accurately estimates larger LAA dimensions is attributed to the elliptical shape of the LAA ostium [19]. While LAAO device sizing is conventionally based on the circular disc of the occluder, the LAA ostium is often oval-shaped with varying degrees of eccentricity, depending on the extent of distortion along its major axis [20]. Conventional 2D-TOE imaging may fail to identify the major axis of an elliptical ostium (Fig. 5), even with views from multiple angles, as shown by Chow et al. [14]. Consequently, undersizing based on 2D TOE would be more pronounced with highly eccentric LAAs. In contrast, 3D assessments from CT consistently identify the major axis of this oval structure, leading to more accurate measurements of the maximal LAA dimensions. The extent of this discrepancy is illustrated in Fig. 5, in which TOE and CT assessments of an LAA in the same patient demonstrate significant differences in LAA dimensions based on 2D and 3D perspectives of an eccentric LAA.

The shorter procedure time associated with preprocedural CT compared with TOE is based on superior CT spatial resolution and more accurate sizing of elliptical LAAs. This leads to more precise preprocedural sizing, a reduced rate of device size change, and improved planning considerations for the LAAO procedure. Preprocedural imaging for LAAO is necessary for excluding patients with contraindication, accurate device sizing, procedural efficiency, and preventing complications from peridevice leakage or device embolization. In addition to the invasiveness of preprocedural TOE, the requirement for fasting, and provision of only 2D assessments, further inaccuracies of LAA measurements can be introduced based on the patient’s volume status as LAA dimensions are highly dependent on preload [21]. The consistent undersizing of LAA dimensions by TOE reflects the limitations of 2D assessments in evaluating elliptical structures and the impact of fasting and low preload states in false reductions in LAA dimensions. This consistent undersizing results in the need for additional time to exchange the deployed suboptimally sized device and resize the LAA intraprocedurally with LAA angiography.

Beyond the inadequacies of TOE, preprocedural CT enhances planning considerations, resulting in shorter procedure times. Noninvasive 3D LAA assessments provide more accurate measurements of landing zones and depths. A 3D reconstructed image also provides an LAA angiogram roadmap, guiding optimal C-arm angulation for transseptal puncture and device implantation [13]. By improving LAAO procedural workflows through enhanced planning considerations from preprocedural CT, the operator can better anticipate optimal transseptal puncture positions and reduce the procedure time.

The results from our study align with those of a recently published study by So et al. [22], in which preprocedural CT for a Watchman LAAO device (Boston Scientific) implantation resulted in shorter procedure times and a reduced likelihood that the device size would need to be changed compared with TOE. Given that cardiac CT has become the gold standard in imaging for planning transcatheter aortic valve implantation (TAVI) procedures [23], and the complex and variable structure of the LAA presents similar challenges to TAVI in terms of device sizing and residual leaks [24], adopting a 3D assessment with CT as the mainstay modality for preprocedural imaging in LAAO procedures may be logical, particularly after the development of new and more accurate LAA measurement parameters such as perimeter-derived diameter [10]. While our study’s consistent findings of more accurate LAA measurements and shorter procedural times with CT support extending the gold-standard use of preprocedural CT from TAVI to LAAO procedures, further randomized studies are required to confirm this conclusion.

Our study has several limitations that must be acknowledged. First, the sample size (n = 64) within our single-center study was small. This small representation prevents generalizability of our results to the wider population. Second, the lack of randomization subjects subjected our study to selection bias when selecting preprocedural imaging modality. This could result in unacknowledged systematic differences between the two cohorts. Third, only Amplatzer Amulet LAAO devices were used, further limiting the generalizability of our findings to LAAO procedures using the same device.

## Conclusions

The planning of LAAO procedures using preprocedural CT was associated with shorter procedure time, lower rate of device size change, and a reduced likelihood of underestimating the maximum landing zone diameter compared with preprocedural TOE.

## Data Availability

Not applicable.

## Abbreviations

AF: Atrial fibrillation
CT: Computed tomography
D: Dimensional
HU: Hounsfield units
ICE: Intracardiac echocardiography
LA: Left atrium
LAA: Left atrial appendage
LAAO: Left atrial appendage occlusion
LOA: Limits of agreement
OAC: Oral anticoagulation
TAVI: Transcatheter aortic valve implantation
TOE: Transesophageal echocardiography

## Data Availability

Data is provided within the manuscript.
